# Regular Physical Activities Inhibit Risk Factors of the Common Cold Among Chinese Adults

**DOI:** 10.3389/fpsyg.2022.864515

**Published:** 2022-05-24

**Authors:** Renjie Tu, Yifan Lu, Kuan Tao

**Affiliations:** ^1^School of Sport Medicine and Physical Therapy, Beijing Sport University, Beijing, China; ^2^School of Sports Engineering, Beijing Sport University, Beijing, China

**Keywords:** Chinese, risk factor (RF), colds, exercise intensity, physical activity

## Abstract

**Background:**

Physical activity (PA) has a significant health impact worldwide and has been linked to a lower risk of the common cold.

**Objective:**

The aim of this study was to estimate the form of PA among Chinese adults and the correlation between PA and number of the common cold in China's eastern, central, and western areas.

**Design:**

A cross-sectional study.

**Setting:**

China's eastern, central, and western regions from 30 November 2020 to 30 March 2021.

**Patients:**

A total of 1,920 healthy participants, who aged over 18 years old, with Internet access, were enrolled, and then self-reported PA behaviors and number of the common cold were collected.

**Measurements:**

The authors calculated preference, intensity, frequency, and duration of PA in Chinese based on gender, age, and broad occupational categories and explored the potential effect between these factors and the common cold.

**Results:**

Approximately 20.4% of participants reported not participating in sports regularly. Except for gender, there were significant differences in PA preference and intensity among the remaining individuals (*P* <0.05). Sixteen common exercises were divided into three intensity levels by the Borg CR10 Scale: low- (5), moderate- (8), and high-intensity exercises (3), and the corresponding intensity, frequency, and duration were computed with significant differences (*P* <0.05). The most popular workouts are “Brisk walking” and “Running.” Age, sex, and occupation had no significant effect on colds (*P* > 0.05). However, intensity shows a U-shaped dose-response relationship with colds, whereas the frequency and duration have an inverse dose-response relationship (*P* <0.05). High intensity combined with high frequency increased colds the most. Nevertheless, non-exercise groups always have the most colds in each comparison.

**Limitations:**

The result may be vulnerable to recall bias.

**Conclusion:**

Intensity showed that U-shape, frequency, and duration showed inverse response to the number of colds last year, but age, sex, and occupation had no significant effects. High intensity and high frequency mixed increased colds the most, regardless of duration.

## Introduction

Physical activity (PA) has a significant impact on global health. PA is considered a principal intervention for primary and secondary disease intervention, and a lack of PA or excessive physical efforts might put you at risk for a variety of diseases (Tiollier et al., 2005; Nieman, 2009; Lee et al., 2012; Durstine et al., 2013). Particularly in the context of the coronavirus disease 2019 (COVID-19) pandemic, some evidence suggested that regular exercise may reduce the risk of infectious diseases such as pneumonia, influenza, and COVID-19 (Ostchega et al., 2000; Pape et al., 2016; Nieman, 2020; Chastin et al., 2021). Regular exercise may also result in higher concentrations of CD4 T cell helpers and salivary immunoglobulin IgA, which may contribute to a rapid and more robust immune response (Chastin et al., 2021; da Silveira et al., 2021). Current guidelines advocate regular, moderate PA to prevent infectious diseases (Campbell and Turner, 2018; Chastin et al., 2021). Individualized exercise prescription may reduce the cost of public health (Bolognese et al., 2020). While many factors can influence PA involvement, previous studies have begun to uncover the impact of gender, age, occupation, and other characteristics. Some have calculated the overall weekly intensity and activity hours by occupation in the United States, as well as the hours spent and energy consumed in PA domains in Canada (Csizmadi et al., 2011; Steeves et al., 2018). Furthermore, data demonstrated a strong correlation between moderate intensity or high frequency and lower risk of colds (Chubak et al., 2006; Nieman et al., 2011). The survey of the daily PA by different classifications and the link between PA and common colds were of far-reaching influence on public health.

However, compared with most of this topic focused on developed countries, only a few studies were conducted in China to investigate the correlation between PA and the aforementioned factors (Jurj et al., 2007; Chen et al., 2015; Zhou et al., 2018; Mao et al., 2020). They have discussed the role of socioeconomic status in PA behaviors and changes in the total amount of PA over 15 years in Chinese. Others have investigated the association between high-frequency leisure-time exercise and the reduction of the common cold, in addition to the gender differences in exercise behavior. However, less research has been conducted to determine Chinese PA preferences and the related intensity, frequency, and duration. The research on the relationship between cold intensity, frequency, and duration and their combined effect on colds was similarly muddled. Precise studies are primarily required based on the assumption of a Chinese study of exercise habits and the dose connection between PA and colds.

Therefore, we aimed to investigate the forms of PA in Chinese adults under different classifications and the correlation between the intensity, frequency, duration of PA, and number of the common cold.

## Methods

### Study Design

This was a cross-sectional study of healthy adults over the age of 18 with an Internet connection. This study used a multistage random sampling method, and the questionnaire was available between 30 November 2020 and 30 March 2021. Participants can respond to the questions on the questionnaire platform (https://www.wjx.cn/jq/96805671.aspx) by scanning the QR code from the experimenter and completing the questions (the entire questionnaire is presented in Appendix). The examiner would exchange contact information on WeChat with the participant when he/she was first recruited. To make sure the recall was accurate, the participant was educated on instructions to inform the examiner on WeChat within 30 min after finishing any personal common exercise. After receiving confirmation from the participant, the examiner would provide them with a QR code to complete the questionnaire. Every participant's IP address and submission time were collected to make sure no one entered it more than once. A total of 30 samples of people were retested for reliability before the formal investigation. This questionnaire was then shared widely across eastern, central, and western China in this way. Participation in the survey was voluntary, and no monetary incentive was offered.

### Variables

The survey captured information across these areas: demographic and occupational information, PA intensity, duration, frequency, PA forms, and number of the common cold. To obtain behaviors before 1 year, data will be collected using a self-reported questionnaire with qualified reliability (with reliability tests shown in the “Results” section).

### Measurement

The common cold was assessed with a single term: “How many times have you had a common cold in the last year?”. Response options included “zero,” “once,” “twice,” “three times,” and “four and more times,” like previous studies (Benseñor et al., 2001; Matthews et al., 2020). Before responding to the number of common colds, we told them the clinical manifestations of common cold, such as nasal stuffiness and discharge, sneezing sore throat, and cough, to help them make a self-diagnosis (Heikkinen and Järvinen, 2003).

The PA intensity was assessed using the Borg CR10 Scale: “How did your feel in general during the last year in physical activity? And how has your heart felt during physical activity over the last year? (heart/muscle/respiratory system).” Participants were questioned about their general/muscle/heart/respiratory system effort feelings within 30 min of confirming completion of any individual common exercise. Response options were on a scale of 1–10, with higher numbers indicating a more tired feeling.

The PA frequency was assessed with a single term: “How about the frequency of doing your common physical activity per week in the last year?” Response options included “zero,” “once or twice,” “three to five times,” “almost every day,” and the answer was analyzed using the mean value of the given range (such as 0, 1.5, 4, and 6 per week, respectively).

The PA duration was assessed from two aspects: “How about the duration of doing your common physical activity in the last year? And how long have you insisted on that? Response options included “0.5 h,” “1 h,” “1.5 h,” and “2 h or longer” (regarded as 2 h). The duration of persistence time varies from 1 month to 1 year. The number of colds was assessed using a single item: “How many colds have you infected in the last year?”, participants can choose from “once,” “twice,” “three times,” or “more than four times (regarded as 4 times).”

The PA forms mainly include the activity area and the type of PA. Activity area options included a park, communal areas, gyms, public roadways, community dedicated sports venues, and personal courtyards. For the type of PA, participants were expected to complete the short format (16 types in total) or the long format if their favorite sport was not available.

### Sample Size

The sample size was determined according to the formula:


(1) $$\begin{array}{r} N={\left(z^{2}\right)}^{*}\left[P\left(1-P\right)\right]/E^{2}\; \end{array}$$

A 95% confidence level (CI) was used in this study so that *Z* was 1.96. *P* was the expected value of the proportion of the target population. In general, the maximum variability value of the sample was 0.5. *E* was the allowable sampling error, usually not <2–3%. Based on the 80% effective sample recovery rate, the number of questionnaires to be issued was as follows:


(2) $$\begin{array}{r} N={\left(1.96^{2}\right)}^{*}\left[0.5\left(1-0.5\right)\right]/0.025^{2}/0.8=1920\; \end{array}$$

### Statistical Analysis

The data were presented with means ± standard deviations (SD) or numbers (proportions). Demographic, age, sex, and occupation factors were assessed using *t*-tests or Wilcoxon rank-sum tests. The difference in CR10 between exercise types and the number of colds at different intensities, frequencies, and durations was compared using the Kruskal–Wallis test and a 95% CI accounted for the number of colds. The odds ratio (OR) was used to measure effect size, an intuitive and commonly used measure in the medical literature (Jia et al., 2017). A multivariable generalized linear model was used to synthesize associations between variables and the number of common colds, producing a forest plot to visually assess the OR and 95% CI. The confirmatory analysis and the factor load of latent variables were performed using AMOS 22, which is shown in the “Results” section. The distribution diagram of colds was plotted using MATLAB in a four-dimensional color map. All analyses of data were performed using IBM SPSS Statistics 21, and the tests of statistical significance were based on two-tailed probability.

### Approvals and Permissions

This study was approved by the Ethics Review Committee of Beijing Sport University (Grant number No. 201906711), and all data collection was anonymous.

### Role of the Funding Sources

This study was supported by grants from the subproject of the National Key Research and Development Program of the Ministry of Science and Technology, China (2018YFC2000603). The funding sources were not involved in the study design, analysis, and writing parts.

## Results

### Participants

**Figure 1:**
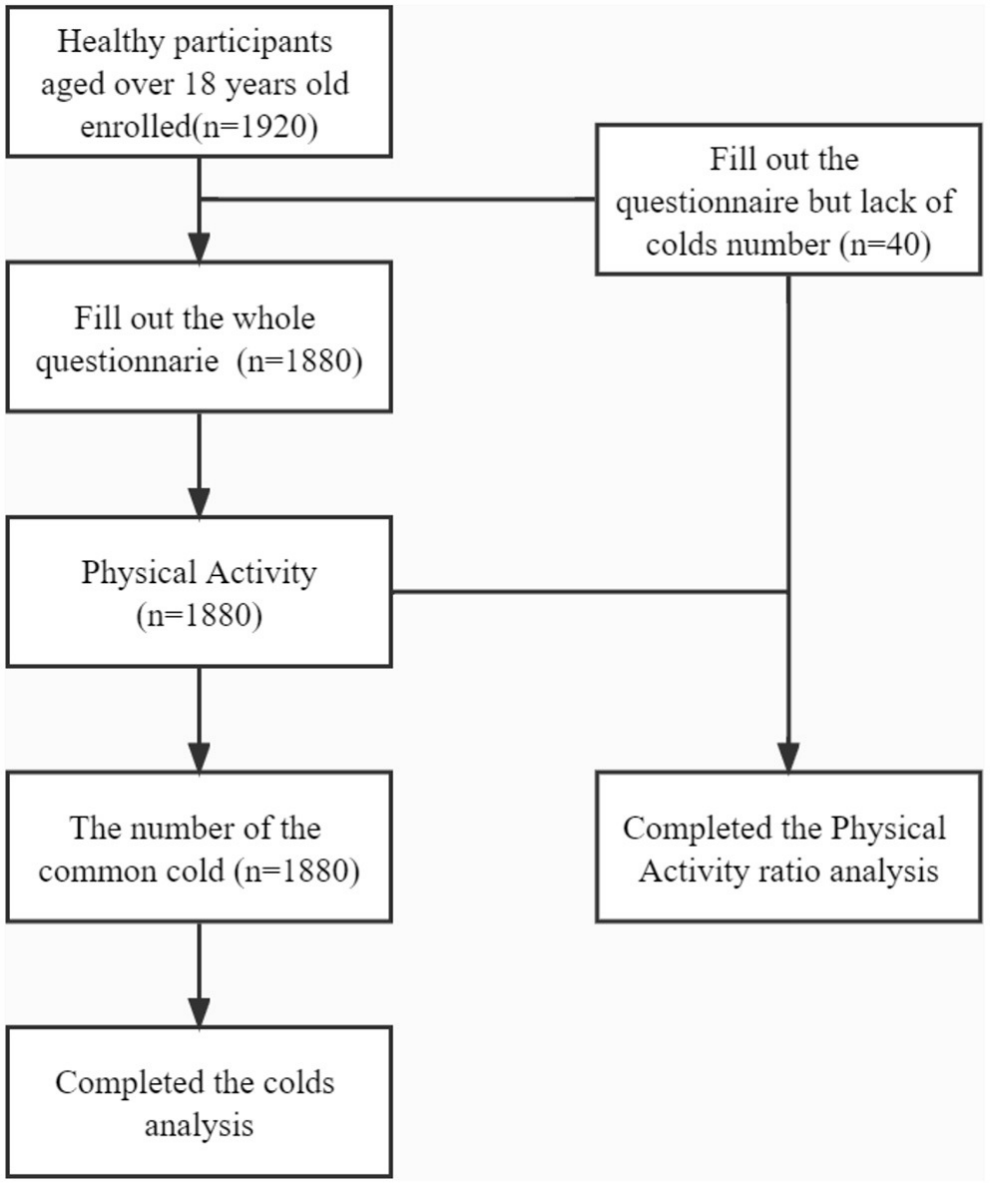
CONSORT diagram of the survey responses.

In total, 1,920 individuals provided consent forms, 1,880 individuals completed the entire questionnaire, and the rest did not complete it because they failed to remember the number of colds. Therefore, these 1,880 individuals were included in further analyses of PA form, exercise intensity, frequency, duration, and colds, and the remaining 40 people were also included in the analysis of the non-exercise ratio (Figure 1).

### Different Groups for PA Preference

Individuals primarily resided in the Central and Eastern Regions of China, which accounted for 76.6% of the sample. Results for different groups of primary and secondary PA forms, intensity, in CR10, and ratio of non-exercise people are presented in Table 1.

**Table 1:** Participant characteristics of reported regarding primary and secondary physical activity (PA) forms, CR10, and nonexercise ratio.

	***n***	**Primary PA, *n***	**CR10**	**Secondary PA, *n***	**CR10**	**Non-exercise**	***P*-value**
**Sex**
Men	839	Brisk walking 346	2.5 ± 1.0	Running 111	3.5 ± 1.1	137	0.361*^S^*
Women	1,081	Brisk walking 436	2.5 ± 1.1	Running 122	3.0 ± 1.1	254	0.197*^C^*
**Age**
0–20	163	Running 62	3.0 ± 1.0	Brisk walking 33	3.0 ± 1.4	30	
21–40	641	Running 122	3.5 ± 1.1	Brisk walking 121	3.0 ± 0.9	165	<0.001*^S^*
41–60	393	Brisk walking 189	2.5 ± 1.2	Others 22	2.5 ± 0.8	85	<0.001*^C^*
61–70	349	Brisk walking 219	2.5 ± 0.9	Square dance 39	2.5 ± 0.9	39	
71 or more	374	Brisk walking 220	2.5 ± 1.1	Square dance 21	2.5 ± 1.0	72	
**Occupation**
Executives	138	Brisk walking 65	2.5 ± 0.9	Resistance training 10	4.5 ± 2.5	20	
Professionals	251	Brisk walking 90	2.5 ± 1.5	Running 31	3.5 ± 1.1	59	
Clerks	54	Brisk walking 17	2.5 ± 1.0	Resistance training 5	4.0 ± 1.9	19	
Business or services	118	Brisk walking 42	2.0 ± 1.0	Running 7	3.5 ± 1.3	41	
Agriculture	135	Brisk walking 75	2.0 ± 1.0	Running 14	2.0 ± 0.7	22	<0.001*^S^*
Operational staff	30	Brisk walking 13	3.0 ± 1.0	Yoga 2	3.0 ± 0.9	8	<0.001*^C^*
Student	474	Running 144	3.5 ± 1.1	Brisk walking 78	3.0 ± 1.3	95	
Unable to classification	158	Brisk walking 51	3.0 ± 1.1	Resistance training 12	4.0 ± 1.6	51	
Soldier	9	Running 2	3.5 ± 0.7			2	
Retiree	553	Brisk walking 350	2.5 ± 0.8	Square dance 50	2.5 ± 0.8	74	

C *Indicates that comparisons of CR10 among groups are significant with P-value*.

S *Indicates that comparisons of PA forms among groups are significant with P-value*.

Approximately 20.4% of participants reported not having a sports habit, with women accounting for a higher proportion of non-exercise (1.44:1). Besides, there was no significant difference in the intensity and preference of exercise between women and men, with the same primary and secondary PA forms (brisk walking and running, *P* = 0.361, *P* = 0.197).

When the participants were divided into ages, the groups aged 21–40 years showed the maximum intensity of exercise. In the primary choice of PA, young individuals chose running as their major PA form, whereas the middle-aged and elderly preferred “Brisk walking” (*P* <0.001). As for the secondary PA form, “Square dance” appeared to be exclusive to the middle-aged, elderly, and no young people (0–40 years old) participated in this form of PA.

The ratio of non-exercise varied in the groups of occupations, and the proportion of non-exercise in “Retirees” and “Executives” was the lowest (13.5%), while among “Students,” “Clerks,” and “Business or services,” the ratio of non-exercise was high (31.5%). Additionally, for the choice of exercise, “Brisk walking” was still the most popular PA form (except among students), and “Resistance training” and “Run” should also be considered. Given that retirees were usually the elderly, the “Square dance” was particularly popular in this group.

### Different Intensities, Frequencies, and Durations of Exercise

Table 2 provides further information on sixteen PA forms. The intensity of exercises was divided into three levels (i.e., low, moderate, and high). The classification was based on the corresponding relationship of rating of perceived exertion (RPE) intensity provided by ACSM, and the conversion formula between RPE and Borg CR10 Scale was provided by “The-Borg-CR-Scales-Folder” (Gunnar Borg, 2010; Medicine, 2018). When the CR10 score of an exercise was ≤ 3, it was classified as “Low intensity,” 3–3.5 were classified as “Moderate intensity,” 4 and above were classified as “High intensity.” The classification results are reported in Table 2. For the frequency of exercise, most exercises were performed 3–4 times per week, apart from “Tai Chi,” which is commonly reported to be performed an average of 4.7 times per week. For the duration of exercise, most people reported 1 h, except for “Tennis,” “Basketball,” “Volleyball,” and “Football,” which appeared to have a longer duration (more than 1.5 h). Due to a lack of sufficient data, “skiing” has been deleted from Table 2.

**Table 2:** Outcome variables from the questionnaire for all the PA forms involved about the CR10, frequency, and duration.

**PA forms**	***n***	**CR10**	**Frequency**	**Duration**	***P*-value**
**Low intensity**					
Square dance	77	2.5 (1.3)	3.8 (1.8)	1 (0.5)	
Tennis	9	2.5 (0.8)	3.6 (2.1)	1.7 (0.4)	
Tai Chi	28	2.5 (0.7)	4.7 (1.9)	1.3 (0.5)	
Brisk walking	782	2.5 (1.3)	3.8 (1.9)	1 (0.5)	
Aerobics	32	2.5 (0.7)	2.9 (1.6)	0.8 (0.3)	
**Moderate intensity**					
Badminton	30	3 (1.3)	3.2 (1.5)	1.2 (0.5)	
Others	71	3 (1.4)	3.3 (2.0)	1 (0.6)	<0.001*^C^*
Run	233	3 (1.3)	3 (1.6)	1 (0.4)	<0.001*^F^*
Basketball	53	3.5 (1.3)	3.1 (1.5)	1.5 (0.4)	<0.001*^D^*
Yoga	31	3.5 (0.9)	3 (1.7)	1 (0.4)	
bicycle	26	3.5 (1.8)	3.5 (1.8)	1 (0.6)	
Swimming	31	3.5 (1.3)	3.6 (2.0)	1.1 (0.5)	
Table tennis	6	3.5 (0.5)	3.5 (1.7)	1.3 (0.4)	
**High intensity**					
Volleyball	5	4 (3.2)	3.3 (2.5)	1.6 (0.5)	
Resistance training	107	4 (1.9)	4 (1.4)	1.3 (0.4)	
Football	7	4 (3.1)	2.2 (1.2)	1.6 (0.3)	
Skiing	1	6	2	4	

C *Comparisons of CR10 among groups are significant with P-value <0.001*.

F *Comparisons of frequency among groups are significant with P-value <0.001*.

D *Comparisons of duration among groups are significant with P-value <0.001*.

### Multivariable Generalized Linear Model

The multivariable generalized linear model with the generalized estimating equation method was used to assess the association between the number of common cold and the rest of the variables contained in Table 1. Results for the number of colds are presented in Table 3.

**Table 3:** The odds ratio with the number of common cold and presented in the forest plot.

**Variables**	**OR (95%CI)**	***P*-value**	**VIF**	**Forest plot**
				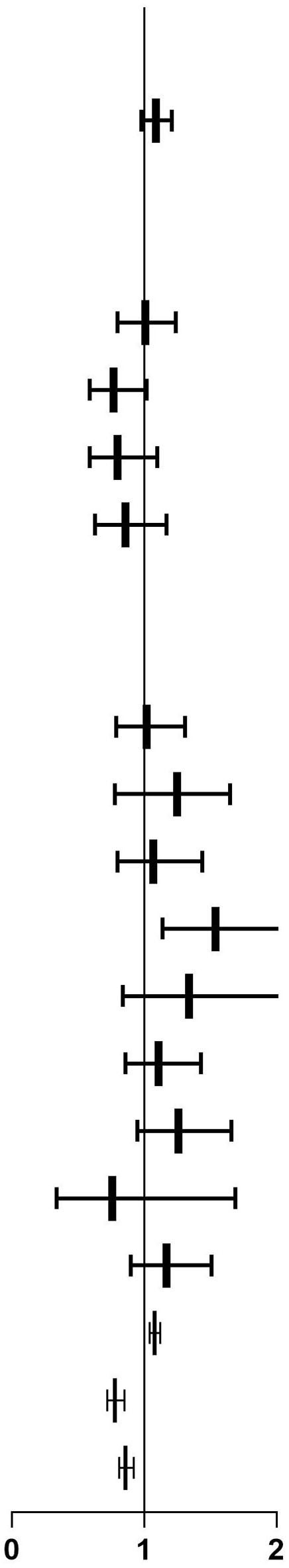
**Sex**			1.02	
Male	Ref	–		
Female	1.09 (0.98–1.21)	0.127		
**Age**			1.26	
18–20	Ref	–		
20–40	1.01 (0.80–1.24)	0.998		
40–60	0.77 (0.59–1.02)	0.072		
60–70	0.80 (0.59–1.10)	0.168		
≥71	0.86 (0.63–1.17)	0.338		
**Occupation**			1.18	
Executives	Ref	–		
Professionals	1.02 (0.79–1.31)	0.869		
Clerks	1.25 (0.78–1.65)	0.512		
Business or services	1.07 (0.80–1.44)	0.638		
Agriculture	1.54 (1.14–2.11)	0.005		
Operational staff	1.34 (0.84–2.15)	0.218		
Student	1.11 (0.86–1.43)	0.420		
Unable to classification	1.26 (0.95–1.66)	0.114		
Soldier	0.76 (0.34–1.69)	0.505		
Retiree	1.17 (0.90–1.51)	0.235		
Intensity	1.08(1.04–1.12)	0.01	2.12	
Frequency	0.78(0.72–0.85)	0.01	2.10	
Duration	0.86(0.81–0.92)	0.01	1.77	

*OR, odds ratio; CI, confidence interval; VIF, variance inflation factor*.

According to Table 3, “Intensity,” “Frequency,” and “Duration” showed a strong correlation with the number of colds with *P*-value <0.05. In contrast, “Sex,” “Age,” and “Occupation” showed some correlation with numbers but with a *P*-value > 0.05. Among the significant variables, “Frequency” and “Duration” presented an inverse dose response while “Intensity” presented a positive dose response. All the variance inflation factors (VIFs) of variables that were <5 demonstrate no high correlation between the independent variables.

### Intensity, Frequency, Duration, and Colds

**Figure 2:**
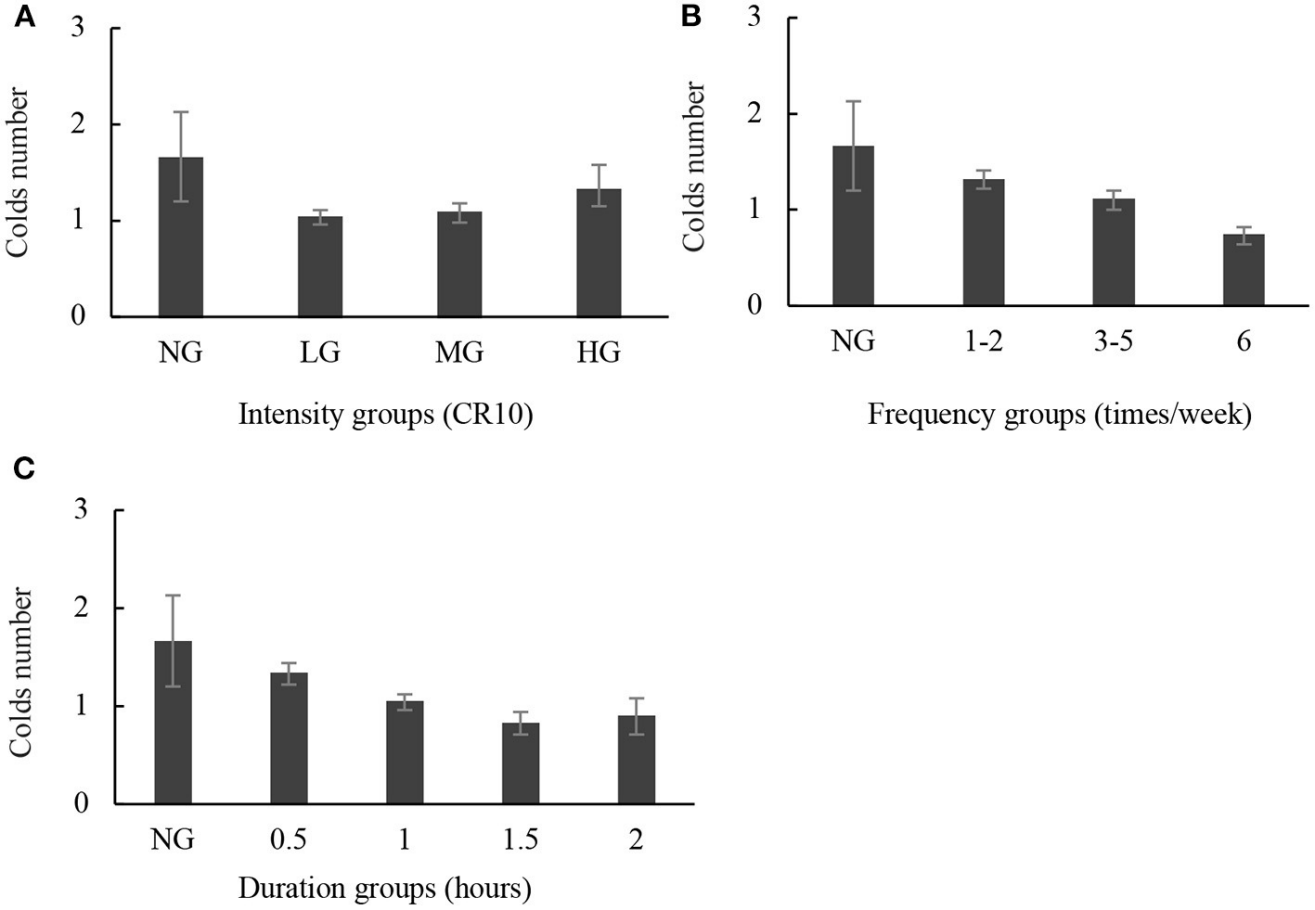
Associations of intensity, frequency, and duration with the number of colds in the last year. **(A)** Intensity with colds, **(B)** frequency with colds, and **(C)** duration with colds. The abscissa represents frequency (times/week) or duration (hours). Data are presented as mean ± 95% CI with *P*-value <0.05.

Figure 2 depicts the specific effect of a single variable on cold (only variables that are significant are included). Unlike the presented in the forest plot, there was a U-shaped dose-response relationship between intensity and colds: the more colds there were (*P* < 0.05), the less exercise there was, but intensity also showed a protective effect when compared with the non-exercise group (NG). The number of colds in the NG was 1.65 (95% CI: 1.17–2.13), whereas the number in the low-intensity group (LG) was merely 1.03 (95% CI: 0.96–1.11). However, in the moderate-intensity group (MG) (1.08, 95% CI: 0.98–1.18) and high intensity group (HG), the number rose (1.32, 95% CI: 1.15–1.58). When examining the association between frequency, duration, and colds, similar patterns and comparable effect sizes were found, with an inverse dose-response in both cases (*P*-value <0.05, Figure 2).

**Figure 3:**
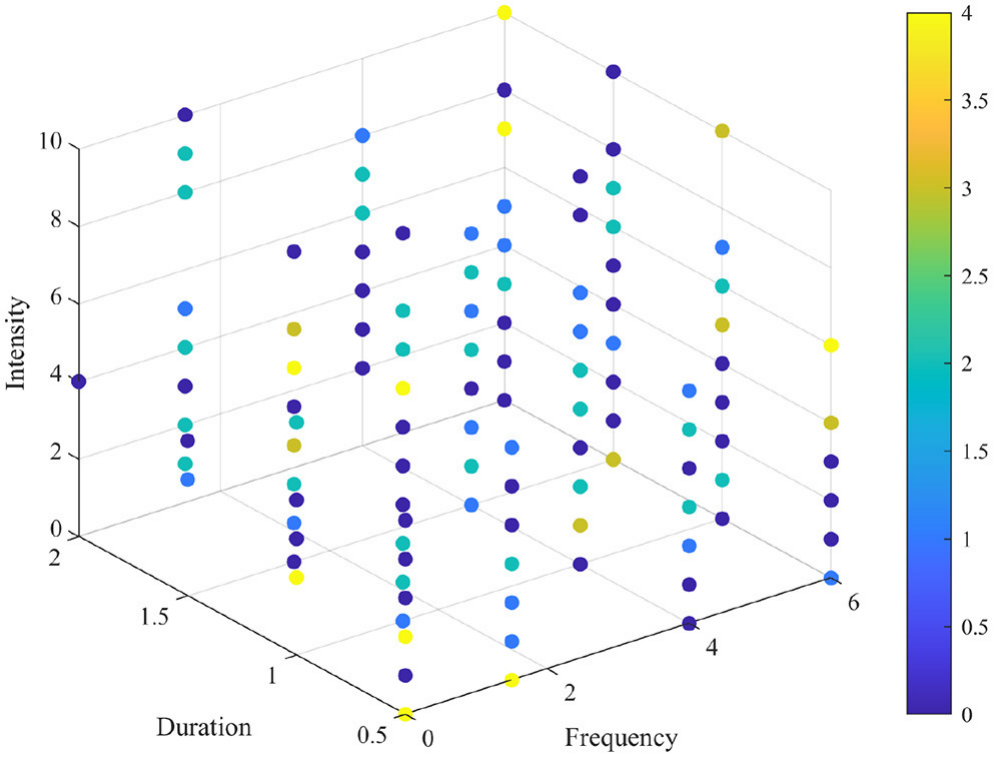
The distribution diagram of colds combined with intensity, frequency, and duration. The color of points means the number of colds in the last year.

Figure 3 shows the distribution diagram of colds when intensity, frequency, and duration were randomly mixed. The color of dots represented the number of colds in the last year, whereas a more yellow and blue color indicated the more and fewer colds, respectively.

Overall, the number of colds in the last year varied significantly in terms of intensity, frequency, and duration. High intensity with high frequency increased the most colds, and NG was always the most affected.

### Reliability and Validity

The reliability and validity of this questionnaire were performed using AMOS 22 and SPSS 21.0. The Cronbach's alpha and the KMO and Bartlett's test coefficient of this questionnaire were 0.62 and 0.802, both were higher than 0.6. Thirty people were required to finish the same questionnaire 2 weeks after they first completed it, and the Pearson correlation coefficient for the question “number of colds in the last year” and “reported intensity of PA in CR10” were 0.812 and 0.885, respectively, both were higher than 0.6. The cumulative variance contribution rate shows 64.3% higher than 50%. The rotational component matrix showed that factor loadings of all variables were higher than 0.5, which means that the reliability and validity were acceptable.

## Discussion

There are several noteworthy findings in this study. Our study supported the opinion that in the generally healthy Chinese people, higher frequency and longer duration of PA were inversely related to the higher number of common colds. Compared with sedentary adults, the high frequency could reduce the severity and number of common cold (Nieman et al., 2011; Zhou et al., 2018). Intensity has a U-shaped dose-response relationship with colds. Previous studies have found that PM_10_ and PM_2.5_ can be associated with reported common cold (Lu et al., 2020). Moderate activity contributed to uniform particle deposition, but intense activity may increase airflow and thus increased inertial impaction, the latter leading to acute effects (Deng et al., 2019). Besides, we discovered that even after long-term strenuous exercise, the number of colds is still lower than that of NG, which complies with Hemilä's findings (Hemilä et al., 2003). We speculated that the reversed trend between intensity with frequency and duration is related to a reduction in lymphocyte concentration (Pedersen and Hoffman-Goetz, 2000), and that the strenuous exercise increased the risk of infection but more frequently moderate activity may not increase (Walsh et al., 2011). When we combined the three, we found that high intensity with high frequency increased colds the most, regardless of duration. Few people considered a specific combination variation, as seen in Figure 2. This demonstrates that the combined effect of different amounts of exercise is indeed of concern, it was difficult to get all participants to comply with the WHO recommendations, such as regular PA for over 150 min of moderate intensity per week (Bull et al., 2020). We offered a more flexible approach, a cold distribution diagram for all age groups. Participants could know their risk of catching a cold and adjust to a safer exercise program based on the distribution of points in this diagram.

Age, sex, and occupation showed no significant effect on colds in this study (*P* > 0.05). Some previous studies found a significant effect between age and colds, but the study was limited to 12 weeks (Nieman et al., 2011), whereas this study investigates colds for the past 1 year. When the measurement time was limited to a short range, the individual difference may result in different findings.

It is known that the intensity and types of PA vary considerably between socioeconomic classes (Chen et al., 2015) and that persons of different classifications or ages also have distinct PA preferences (Holtermann et al., 2012). However, only a few studies, such as ours, have counted Chinese PA preferences and intensity across ages and occupations in Table 1. We discovered that most people focus on the choice of brisk walking or running, implying that researchers need to pay more attention to these high-frequency exercises. Besides, they favored moderate and low intensity to high intensity. These findings can guide our actions when the purpose of research is to be more relevant to daily life.

According to Table 1, we found that there was no significant difference in PA preference or intensity across gender groups. However, there was a correlation between different occupations and ages. However, the observed ratio of non-exercise in some previous studies was even higher than in our studies. They claimed ~7.4–23.6% of individuals engaged in physical activities in addition to their professional duties, even though the result was much lower than the national average in 2010 (Ng et al., 2009; Chen et al., 2015). The lack of consistency in the ratio of PA may come from the advancement of social economics and the differences in crowd choice. People who were less educated of lower socioeconomic status may have limited access to exercise or awareness of the positive health consequences of PA (Parks et al., 2003; Wilson et al., 2004; Gidlow et al., 2006; Kamphuis et al., 2008; Beenackers et al., 2012; El-Sayed et al., 2012); therefore, these findings may lead to a new trend.

The exercise intensity of young people aged 0–40 years was substantially higher than that of those over 40 years with respect to CR-10. The former reached a peak number of 3.5 in CR-10, while the latter barely attained 2.5. As for the occupation classification, the reported ratio of non-exercise in executives, students, and retirees was lower than in others, probably due to their high social status or education level, giving them more free time to exercise. The intensity was universally selected at low or moderate intensity, particularly lower in individuals with a high level of occupational activity (such as agriculture, business, or services). These subtle differences between age and occupations need to be noticed when designing sports interventions.

Second, each type of exercise has different physiological effects (Woolf-May et al., 2000; Finucane et al., 2010; Koh et al., 2018; Chiang et al., 2019), and even for the same exercise, there are different effects at different intensities (Asikainen et al., 2003; Cho et al., 2018). Besides, frequency and duration are also important factors (Woolf-May et al., 1999), so we calculated the intensity, frequency, and duration of PA based on the preferences of the population, as discussed in the previous study (Branco et al., 2021). Despite the fact that football accounts for a 51% share of the global sports market (Yam et al., 2020), the most popular PA forms reported were “Brisk walking” and “Running,” followed by “Resistance Training” and “Square Dance.” Furthermore, ~60% of individuals experienced low-intensity PA, and just 7.6% reported high intensity. The intensity used in research tends to be extremely high at times (Asikainen et al., 2003; Krustrup et al., 2009; Papp et al., 2016; Kuo et al., 2018), which is far distant from the reality of Chinese daily exercise. It is unclear whether the physiological effect can be well-maintained if the interventions performed exercise routines that people would not ordinarily do.

These findings have other clinical and public health implications and are more than just a snapshot of the current situation, which serves as a foundation for future research.

However, we must recognize the several shortcomings of this study. First, intensity, colds, and other variables were counted based on self-report, rather than exact laboratory testing, and the results may be vulnerable to recall bias. However, people perform PA and perceive physical health as subjective behavior. It is the subjective symptoms rather than positive medicine tests to affect the performance of PA. Also, self-diagnosis was easy because the clinical presentation of a cold was so typical (Hemilä et al., 2003). Second, we counted PA behaviors, yet there were additional characteristics that were not taken into consideration, for instance, genetic disorders, the influence of the COVID-19 epidemic, and the health of those in the vicinity. Identifying specific groups that may benefit from precise exercise prescription would be an investigation objective in the future, under the premise of a more precise investigation of PA behaviors to explore if, or to what extent, PA can affect the health of the general population.

## Conclusion

First, our study reveals that ages and occupational categories have a strong association with PA, including PA preference and the corresponding intensity, except for gender classification. Second, we divided Chinese people's 16 common exercises into three levels based on their intensity on Borg CR10 Scale and presented related frequency and duration. Besides, we found that after introducing PA variables, age, sex, and occupation had no significant effect on colds (*P* > 0.05). However, the intensity has a U-shaped dose-response relationship with colds, whereas frequency and duration have an inverse dose–response relationship. High intensity combined with high frequency increased the colds most.

## Data Availability Statement

The raw data supporting the conclusions of this article will be made available by the authors, without undue reservation.

## Ethics Statement

The studies involving human participants were reviewed and approved by Sports Science Experimental Ethics Committee of Beijing Sport University. The patients/participants provided their written informed consent to participate in this study.

## Author Contributions

RT provided original article writing. YL provided ideas for experimental design. KT assisted in the revision and guidance of the article. All authors contributed to the article and approved the submitted version.

## Funding

This study was supported by grants from the National Key Research and Development Program of the Ministry of Science and Technology, China (2020YFF0304700 and 2018YFC2000603).

## Conflict of Interest

The authors declare that the research was conducted in the absence of any commercial or financial relationships that could be construed as a potential conflict of interest.

## Publisher's Note

All claims expressed in this article are solely those of the authors and do not necessarily represent those of their affiliated organizations, or those of the publisher, the editors and the reviewers. Any product that may be evaluated in this article, or claim that may be made by its manufacturer, is not guaranteed or endorsed by the publisher.
