# Supplementary material for: Regular Physical Activities Inhibit Risk Factors of the Common Cold Among Chinese Adults
Source: Front Psychol. 2022 May 24;13:864515. doi: 10.3389/fpsyg.2022.864515 (PMC9171135; doi:10.3389/fpsyg.2022.864515)
Supplement: Supplementary file 1 [file Data_Sheet_1.DOCX]

# Appendix

## Physical activity and common cold questionnaire

1: Please add your gender

1. Male
2. Female

2: Please add your age

1. 18-20
2. 21-40
3. 41-60
4. 61-70
5. 71 or more

3: Please add your province of residence


4: Please add your education background

1. Junior high school and below
2. High school
3. Secondary technical school
4. Junior college
5. Undergraduate
6. Graduate
7. PhD

5: Please add your occupation background

1. Executives
2. Professionals
3. Clerks
4. Business or services
5. Agriculture
6. Operational staff
7. Student
8. Unable to classification
9. Soldier
10. Retiree

6: How about the frequency of doing your common physical activity peer week in the last year?

1. Zero
2. Once or twice
3. Three to five times
4. Almost every day

7: What kind of physical activity do you often choose?

1. Brisk walking
2. Run
3. Swimming
4. Basketball
5. Badminton
6. Square dance
7. Tai Chi
8. Table tennis
9. Skiing
10. Volleyball
11. Football
12. Table tennis
13. Resistance training
14. Yoga
15. Training Aerobics
16. Bicycle
17. Others

8: How about the duration of doing your common physical activity in the last year?

1. 0.5 hour
2. 1 hour
3. 1.5 hours
4. 2 hours or longer

9: How long have you insisted on that?

1. Within two months
2. Two to three months
3. Three to four months
4. Four to six months
5. Six months to a year
6. Others

10: How many times have you had a common cold in the last year?

1. zero
2. once
3. twice
4. three times
5. four and more times

11: Where is your activity area?

1. park
2. communal areas
3. gyms
4. public roadways
5. community dedicated sport venues
6. personal courtyard

12: How did your feel in general during physical activity over last year?

1. 0 Nothing at all
2. 1 Very weak
3. 2 Weak
4. 3 Moderate
5. 4
6. 5 Strong
7. 6
8. 7 Very strong
9. 8
10. 9
11. 10 Extremely strong

13: How has your heart felt during physical activity over the last year?

1. 0 Nothing at all
2. 1 Very weak
3. 2 Weak
4. 3 Moderate
5. 4
6. 5 Strong
7. 6
8. 7 Very strong
9. 8
10. 9
11. 10 Extremely strong

14: H How has your muscles felt during physical activity over the last year?

1. 0 Nothing at all
2. 1 Very weak
3. 2 Weak
4. 3 Moderate
5. 4
6. 5 Strong
7. 6
8. 7 Very strong
9. 8
10. 9
11. 10 Extremely strong

15: How has your respiratory system felt during physical activity over the last year?

1. 0 Nothing at all
2. 1 Very weak
3. 2 Weak
4. 3 Moderate
5. 4
6. 5 Strong
7. 6
8. 7 Very strong
9. 8
10. 9
11. 10 Extremely strong
